# Supplementary material for: Usefulness of serial post-systolic shortening by speckle tracking echocardiography to predict major adverse cardiovascular events and segmental function improvement after acute myocardial infarction
Source: PLoS One. 2020 Dec 31;15(12):e0244589. doi: 10.1371/journal.pone.0244589 (PMC7775100; doi:10.1371/journal.pone.0244589)
Supplement: Attachment — Submitted filename: response to Reviewer 2.docx [file pone.0244589.s003.docx]

Reviewer #1: The author addressed some of the concerns in my review, but there are still some issues that need to be further discussed.

1. There is a parameter called time to peak, which should be provided with strain and strain rate by the software. It could make the manuscript more solid if the author adds that parameter as well.
**Ans:** Mechanical dispersion measured as standard deviation (SD) of time from onset Q in electrocardiography to peak negative longitudinal strain from 18 segments was added.

2. In this study, 2 patients had IRA revascularization before 3 months after admission and the other 2 MACE events before 3 months. They did not have PSS at 3rd month. The author stated that PSS at 3rd month is independently predictive of IRA revascularization and MACE. These 5 patients should be excluded from the research.
**Ans:** We did not excluded these 5 patients because this initial study is designed to repeat echo studies including strain and strain rate in post-AMI patients to see if any parameter could predict MACE or IRA revascularization, not just focus on echo parameter at 3^rd^ months. These 5 patients did not have PSS at 3^rd^ month, and they are excluded when we calculated statistical analysis.

3. Is it possible to reanalyzed the recorded echo imaging using the 17 segment model? According to my knowledge, EchoPAC provided a bullseye plot at least for longitudinal strain, and time to peak for strain and strain rate.

**Ans:** Sorry, we could not reanalyze the strain and strain rate using 17 segment model. Most raw data images were lost because the hard drive storage got broken and new version of EchoPAC was installed in Dec 2017.

Reviewer #2: The authors did attempt to correct the calculation errors noted in Table 1. However, they have mistakenly placed the recalculated percentage values in the wrong column of the Table. Please revise.
(In the Event-free group, 32 out of 72 patients (44.4%) had LAD being the culprit vessel. In the Event group 14 out of 22 patients (63.6%) had LAD being the culprit vessel.)

**Ans:** Sorry. I corrected it. Thank you.
